# Bone marrow stromal cell‐derived exosomes improve oxidative stress and pyroptosis in doxorubicin‐induced myocardial injury in vitro by regulating the transcription of GSDMD through the PI3K‐AKT‐Foxo1 pathway

**DOI:** 10.1002/iid3.810

**Published:** 2023-03-27

**Authors:** Hong Zeng, Yong Yang, Fangfang Tou, Yuliang Zhan, Songtao Liu, Pengtao Zou, Yanmei Chen, Liang Shao

**Affiliations:** ^1^ Department of Cardiology, Jiangxi Provincial People's Hospital The First Affiliated Hospital of Nanchang Medical College Nanchang Jiangxi People's Republic of China; ^2^ Department of Cardiology, Union Hospital, Tongji Medical College Huazhong University of Science and Technology Wuhan Hubei People's Republic of China; ^3^ Jiangxi Provincial People's Hospital The First Affiliated Hospital of Nanchang Medical College Nanchang Jiangxi People's Republic of China

**Keywords:** bone marrow stromal cells, exosomes, Foxo1, GSDMD, oxidative stress, PI3K‐AKT, pyroptosis

## Abstract

**Objectives:**

Doxorubicin (DOX) can contribute to severe myocardial injury, and bone marrow stromal cells (BMSC)‐exosomes (Exos) improves acute myocardial infarction. Hence, this research investigated whether BMSC‐Exos alleviated DOX‐induced myocardial injury.

**Methods:**

BMSC‐derived Exos were isolated and identified, and the optimal concentration of DOX was confirmed. H9C2 cells were treated with DOX and BMSC‐Exos or in combination with the protein kinase B (AKT) inhibitor. Reactive oxygen species (ROS) and JC‐1 were detected to assess oxidative stress (OS) and mitochondrial membrane damage, respectively. In addition, the expression of pyroptosis‐related molecules was measured. The expression of phosphatidylinositol 3 kinase (PI3K)‐AKT pathway‐related proteins and the phosphorylation and acetylation of forkhead box O1 (Foxo1) in the cell nucleus and cytoplasm were tested. Last, interactions between Foxo1 and gasdermin D (GSDMD) were assessed.

**Results:**

BMSC‐Exo treatment increased viability and mitochondrial membrane potential and reduced lactic dehydrogenase release and ROS levels in DOX‐treated H9C2 cells. Furthermore, the addition of BMSC‐Exos suppressed DOX‐induced activation and upregulation of NLRP3 and apoptosis‐associated speck‐like protein containing A CARD (ASC) and in vitro cleavage of caspase‐1, GSDMD, interleukin (IL)‐1β, and IL‐18 proteins. Additionally, BMSC‐Exo treatment enhanced the expression of phosphorylated (p)‐PI3K, p‐AKT, and p‐mTOR in DOX‐treated H9C2 cells and the levels of phosphorylated Foxo1 in the cytoplasm of DOX‐treated H9C2 cells. Foxo1 was enriched in the promoter region of GSDMD. Moreover, the AKT inhibitor API‐2 annulled the effects of BMSC‐Exos on OS, pyroptosis, and Foxo1 phosphorylation in DOX‐treated H9C2 cells.

**Conclusions:**

BMSC‐Exos phosphorylated Foxo1 and inactivated Foxo1 transcription via the PI3K‐AKT pathway to diminish GSDMD expression, thus restraining DOX‐induced pyroptosis and OS of myocardial cells.

## Graphical abstract

Bone marrow stromal cells ‐derived exosomes phosphorylate forkhead box O1 (FoxO1) by activating the phosphatidylinositol 3 kinase (PI3K)‐protein kinase B (AKT) signaling pathway to repress gasdermin D (GSDMD) transcription, thus ameliorating doxorubicin (DOX)‐induced oxidative stress and pyroptosis in cardiomyocytes.

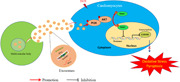

## 1 INTRODUCTION

Isolated from streptomyces peucetius, doxorubicin (DOX) is an effective antibiotic in treatment of various tumors.^1^ However, a large dosage use of DOX results in hard‐to‐treat injuries in the myocardium, which may bring about heart dysfunction, severe heart failure, and even death, especially among children and adolescents.^2^ DOX was known to be responsible for myocardial cell death in terms of multiple cellular processes such as autophagy, ferroptosis, necroptosis, and apoptosis.^3^ Oxidative stress (OS) and pyroptosis are two crucial mechanisms of DOX‐induced myocardial injury.^4^, ^5^, ^6^ Nevertheless, the currently accepted therapeutic methods for DOX‐induced myocardial injury are limited and none of them obtain completely satisfactory efficacy.^7^ To advance the treatment of DOX‐induced myocardial injury, it is necessary to probe the molecular mechanism underlying OS and pyroptosis in the myocardium.

Exosomes (Exos) are 30–150 nm extracellular vesicles produced by cells and play crucial roles in cell‐to‐cell communication, normal life activities, and disease diagnosis and treatment.^8^, ^9^ Exos have been documented to confer protection against OS and pyroptosis in diseases.^10^, ^11^ Bone marrow stromal cells (BMSCs) are a heterogeneous mixture of variable subpopulations with functional and molecular properties^12^ and Exos derived from these cells were known to manipulate the function and processes of different cells to be implicated in the treatment of diseases.^13^, ^14^ It was reported that BMSC‐derived Exos participate in the alleviation of OS in ischemic stroke and function in reducing inflammasome‐associated pyroptosis.^15^, ^16^ More importantly, previous research stated that BMSC‐Exos improve acute myocardial infarction.^17^

As reported, the phosphatidylinositol 3 kinase (PI3K)‐protein kinase B (AKT) pathway participates in the repressive effects of BMSC‐Exos on cell apoptosis in myocardial ischemia–reperfusion injury.^18^ The PI3K‐AKT was a vital pathway regulating cellular processes such as cell survival, division, and differentiation.^19^ Of note, the PI3K‐AKT pathway was displayed to protect against DOX‐induced cardiotoxicity.^20^ Meanwhile, the PI3K‐AKT pathway can phosphorylate forkhead box O1 (Foxo1), one of the downstream molecules of this pathway.^21^, ^22^ Foxo1 is an important molecule regulating transcription and antioxidative enzymes.^23^ Additionally, an earlier study uncovered that Foxo1 activation participates in alleviating DOX‐induced cardiac dysfunction.^24^ Besides, gasdermin D (GSDMD), a pyroptosis execution protein, was revealed to mediate pyroptosis in spinal cord injury and can be activated by Foxo1.^25^, ^26^ It was illustrated that GSDMD was capable of binding to DOX and mediating pyroptosis of myocardial cells.^27^

Based on all these studies, we proposed a hypothesis that BMSC‐Exos modulate GSDMD levels through the PI3K‐AKT‐Foxo1 pathway to affect OS and pyroptosis in DOX‐induced myocardial injury. Thereafter, we conducted a set of experiments to confirm this hypothesis, thus providing novel promising therapeutic targets for DOX cardiotoxicity.

## 2 MATERIALS AND METHODS

### 2.1 Cell culture and identification

Rat BMSCs and H9C2 cells were obtained respectively from the previous study in our lab and American Type Culture Collection (ATCC) and cultured in a Dulbecco's Modified Eagle Medium (DMEM; Thermo Fisher Scientific) which contained 10% fetal bovine serum (FBS), streptomycin (100 g/mL), and penicillin (100 units/mL) under a condition of 37°C and 5% CO_2_.

BMSCs at the third passage and with 90% confluence were seeded in six‐well plates at 1 × 10^5^ cells/mL. Cells were induced for differentiation separately with adipogenic differentiation and osteogenic differentiation mediums. After 14 days, cells were stained with Oil Red O staining (Sigma‐Aldrich) for detection of adipogenic differentiation of BMSCs. Alizarin Red (Sigma‐Aldrich) was then utilized for staining to measure the osteogenic differentiation of BMSCs 21 days later.

BMSCs underwent flow cytometry analysis and probed for 30 min with anti‐CD29‐fluoresceinisothiocyanate (FITC), anti‐CD90‐FITC, anti‐CD44‐FITC, anti‐CD45‐FITC, and anti‐CD34‐FITC. Subsequently, goat anti‐rabbit immunoglobulin G (IgG, ab6717, 1:1000; Abcam) secondary antibodies coupled with FITC fluorochrome were added and cultured for 30 min. A flow cytometer was employed for measurement.

### 2.2 Isolation and identification of Exos

Exos were isolated from BMSC supernatant and incubated for 36 h in a serum‐free medium to avoid the influence of FBS on Exos. After removal of cell debris and related apoptotic debris with differential centrifugation, concentrated cell supernatants were filtered and further characterized by density gradient centrifugation with iodixanol (OptiPrep™; Axis‐Shield).^28^ Electron microscope observation was also conducted: Exo resuspensions were precipitated with ultracentrifugation and subjected to 1‐h fixing (2% paraformaldehyde and 2.5% glutaraldehyde) at 4°C, three washes with phosphate‐buffered saline (PBS, 15 min/time), 1.5 h fixing with 1% osmic acid, and three rinses with PBS (15 min each time) in sequence. Next, after dehydration with graded ethanol and overnight soaking and embedding in epoxy resin, samples underwent 24‐h aggregation at 35°C, 45°C, and 60°C. Samples were then cut into ultrathin sections and treated with lead–uranium double staining, followed by observations under a transmission electron microscope (JEM‐1011; JEOL) at 80 kV voltage of acceleration. Side‐mounted Camera‐Megaview III (Soft Imaging System) was used for photography. Each experiment was repeated three times.

Exos were also identified with western blot: Alix, CD63, Golgi Matrix Protein 130 (GM130), cytochrome *c*, and calnexin expression was tested in BMSCs and BMSC‐derived Exos.

### 2.3 Nanoparticle tracking analysis (NTA) to observe the concentration and size distribution of Exos

NTA was utilized to measure the size and concentration of Exos: Exo samples were resuspended in PBS and diluted 500 times with Milli‐Q water. Afterward, diluted Exo samples were injected into the sample room of NanoSight LM10 (Malvern Panalytical Ltd.) with a sterilized syringe to ensure no air bubbles till the sample room was full. NanoSight LM10 was equipped with a 640‐nm laser and a fluoroelastomer Oring (Viton™; DuPont). NanoSight version 2.3 software (Malvern Panalytical Ltd.) was applied for video analysis, and the motion trails of particles were recorded with the gain value of 6.0 and the threshold value of 11 for all samples. Figures of concentration and size distribution of the diluted samples were output and the Exo concentration of the original liquid was figured out according to the dilution ratio. The experiment was repeated three times.

### 2.4 Laser scanning microscope (LSM) to examine the uptake of Exos by H9C2 cells

BMSCs were seeded in the 24‐well plates at 1 × 10^5^ cells/well, followed by culture in a 37°C incubator containing 5% CO_2_. Subsequent to 24 h, BMSCs were washed three times with PBS and then cultured for 24 h in a low glucose‐DMEM. Subsequently, Exos were isolated from transfected BMSCs and then labeled with a PKH‐67 fluorochrome kit (MINI67‐1KT; Sigma‐Aldrich). Next, Exos were quantified with a bicinchoninic acid (BCA) kit (Pierce). Thereafter, 1 mL Diluent C solution was added to 200 μg Exos and 4 μL PKH67 fluorochromes, respectively. These two solutions were gently and evenly mixed for 5 min, after which the mixture underwent 2‐h centrifugation at 4°C and 100,000*g*. The supernatants were then discarded and rinsed twice with PBS. Last, after 2 h of centrifugation at 100,000*g* (4°C), the labeled Exos were collected.

H9C2 cells (5 × 10^4^ cells/well) were spread in the 24‐well plates, incubated overnight, and then cultured for 4 h with PKH67‐labeled Exos at 37°C with 5% CO_2_, with an equal volume of PBS as the blank control. H9C2 cells were then fixed in methanol and arranged into PBS and Exo groups. LSM 780 (Carl Zeiss) was applied to evaluate the internalization of BMSC‐Exos by H9C2 cells.

### 2.5 Western blot

H9C2 cells were lysed in enhanced radio‐immunoprecipitation assay cell lysis buffer (BOSTER) with protease inhibitor, followed by measurement of protein concentration with the BCA quantitation kit (BOSTER). Proteins were separated with 10% sodium dodecyl sulfate‐polyacrylamide gel electrophoresis, after which they were transferred to polyvinylidene fluoride membranes and sealed for 2 h in 5% bovine serum albumin (BSA) at room temperature to block nonspecific binding. The membranes were supplemented respectively with diluted primary antibodies against Alix (ab275377, 1:1000; Abcam), CD63 (PA5‐92370, 1:1000; Thermo Fisher Scientific), GM130 (PA5‐95727, 1:2000; Thermo Fisher Scientific), cytochrome *c* (ab133504, 1:1000; Abcam), calnexin (ab133615, 1:1000; Abcam), nucleotide‐binding oligomerization domain, leucine rich repeat and pyrin domain containing 3 (NLRP3, ab263899, 1:1200; Abcam), apoptosis‐associated speck‐like protein containing A CARD (ASC, 307560, 1:1000; Abcam), cleaved caspase‐1 (89332S, 1:1000; Cell Signaling Technologies [CST]), interleukin (IL)‐18 (67775S, 1:1000; CST), cleaved IL‐1β (63124S, 1:1000; CST), active N‐terminal fragment of GSDMS (NT‐GSDMD, 10137S, 1:1000; CST), phosphorylated‐phosphatidylinositol 3‐kinase (p‐PI3K, ab278545, 1:1000; Abcam), p‐protein kinase B (p‐AKT, 4060T, 1:1000; CST), total‐AKT (4685S, 1:1000; CST), p‐mechanistic target of rapamycin kinase (p‐mTOR, ab109268, 1:1000; Abcam), p‐Foxo1 (ab259337, 1:1000; Abcam), acetyl‐Foxo1 (PA5‐104560, 1:1000; Thermo Fisher Scientific), and β‐actin (ab8226, 1:2500; Abcam) for overnight probing at 4°C. Subsequent to washing, the membrane was probed for 1 h with horseradish peroxidase‐labeled secondary antibodies at room temperature. Electrogenerated chemiluminescence (ECL) working solution (Millipore) was utilized to incubate with the membrane for 1 min, after which the redundant ECL reagents were removed and the membrane was sealed with plastic wrap and then exposed for 5–10 min with X‐ray film in a dark box, followed by developing and fixing. ImageJ analysis software (National Institutes of Health [NIH]) was applied to quantify the gray scales of protein bands in western blot figures. β‐actin was regarded as the internal reference. Each experiment was repeated thrice.

### 2.6 Cell counting kit (CCK)‐8 assay

CCK‐8 (#A311‐02‐A; Vazyme Bio‐technology Co., Ltd.) was adopted for cell viability detection in simple and efficient manners as instructed in the manuals of the manufacturer. Briefly speaking, after 24‐h DOX treatment (0.5–20 μM), H9C2 cells were treated for 1 h with CCK‐8 solution. A microplate reader (Imark‐22353; Bio‐Rad) was used to measure the optical density (OD) value at 450 nm. Cell viability = OD value of each group/OD value of the control group × 100%. The experiment was repeated independently thrice.

### 2.7 Lactic dehydrogenase (LDH) release test

DOX (0.5–20 μM) was utilized to stimulate H9C2 cells, after which LDH levels in cell supernatants were tested with an LDH cytotoxicity analysis kit (#C0016; Beyotime) per the protocols of the manufacturer. To assess the LDH levels in cell supernatants, the supernatants of H9C2 cells were transferred to a 96‐well plate and each well was added with 60 μL testing solution. Subsequent to 30‐min incubation avoiding light at room temperature, the OD values were determined. The ratio of the OD value to that in the control group was considered the change of multiple. Each experiment was repeated thrice independently.

### 2.8 Determination of intracellular reactive oxygen species (ROS)

After grouping, the 20,70‐dichlorofluorescein diacetate (DCFH‐DA) method was employed for assessment of intracellular ROS levels with a ROS detection kit (#S0033S; Beyotime). In a word, H9C2 cells were stained for 30 min in the dark with DCFH‐DA. Next, a fluorescence microscope was used to monitor cells, and ImageJ software (NIH) to examine the fluorescence intensity of ROS.

### 2.9 JC‐1 mitoscreen assay

Mitochondrial damage was tested with a JC‐1 detection kit (#C2006; Beyotime) in light of instructions from the manufacturer. Cells were incubated with JC solutions and then analyzed with the fluorescence microscope. ImageJ software was applied to quantify the green and red fluorescence intensities in three random fields of view, with the mean value obtained. JC‐1 fluorescence intensity = green fluorescence intensity (mean)/red fluorescence intensity (mean). Three fields of view were randomly selected for each group and the experiment was repeated thrice independently.

### 2.10 Immunofluorescence

After centrifugation, cells were supplemented in fixing agents and permeabilization solution (MultiSciences). H9C2 cells were subject to incubation with 5% BSA, co‐incubation with primary antibodies (NLRP3 and caspase‐1), and overnight probing with secondary antibodies at 4°C. H9C2 cells were then stained for 10 min with 4ʹ,6‐diamidino‐2‐phenylindole (#S0033S; Beyotime). Antifluorescence quenching sealing tablets (#S2100; Solarbio) were utilized for sealing, and a fluorescence microscope (DFM‐90C; Shanghai Cai Kang Optical Instrument Co., Ltd.) was adopted to capture representative images. The green fluorescence intensity in three random fields of view of each group was quantified with ImageJ software and the mean value was figured out. For each experiment, three replicates were conducted.

### 2.11 Enzyme‐linked immunosorbent assay (ELISA)

Contents of IL‐18 (ab213909) and IL‐1β (ab255730) in cell supernatants were detected as directed in the instructions of the manufacturer.

### 2.12 Dual‐luciferase reporter gene assay

The binding site of Foxo1 to the promoter of GSDMD was predicted through the Jaspar database (https://jaspar.genereg.net/). According to the predictive results, wild type sequence (wt‐GSDMD) and mutation sequence (mut‐GSDMD) of the binding site were designed, synthesized, and then respectively inserted into luciferase reporter gene vectors (pGL3‐Promoter), followed by respective co‐transfection with OE‐NC or OE‐Foxo1 into 293T cells. After 48‐h culture, Firefly luciferase activity and Renilla luciferase activity (transfected with phRL‐TK vectors) were measured with luciferase activity detection kits. Renilla luciferase activity was regarded as the internal reference and the ratio of Firefly luciferase activity to Renilla luciferase activity was the relative activity of luciferases. The experiment was repeated three times.

### 2.13 Chromatin immunoprecipitation (ChIP) assay

Subsequent to 4% methanol (final concentration: 1%) treatment, H9C2 cells underwent ultrasonication, after which anti‐Foxo1 (ab179450, 1:30; Abcam) and Foxo1‐GSDMD promoter were supplemented for mutual binding. After complete binding through overnight incubation at 4°C, Protein A Agarose/SaLmon Sperm DNA was added to bind to and precipitate Foxo1 antibody‐Foxo1‐GSDMD promoter complexes. The complexes were washed to remove nonspecific bindings and eluted to obtain the enriched Foxo1‐GSDMD promoter complexes. Subsequent to 5‐min centrifugation at 12,000*g*, the supernatant was discarded, and nonspecific complexes were washed. Samples were subjected to overnight de‐crosslinking at 65°C. Phenol/chloroform was used to extract, purify, and recycle DNA fragments. The enriched fragments of GSDMD promoters were purified, followed by polymerase chain reaction, with IgG (ab109489, 1:100; Abcam) as the negative control. The experiment was repeated thrice.

### 2.14 Statistical analysis

Data were statistically analyzed with GraphPad prism8 software and presented as mean ± standard deviation. After normality analysis of data, the two‐tailed Student's *t* test was adopted for comparisons between two groups and one‐way analysis of variance for those among multiple groups (except for the experimental groups tested by two‐way analysis of variance as per special instructions). Tukey's test was applied for post hoc multiple comparisons. Differences were statistically significant at *p* < .05.

## 3 RESULTS

### 3.1 The isolation and identification of BMSCs

**Figure 1:**
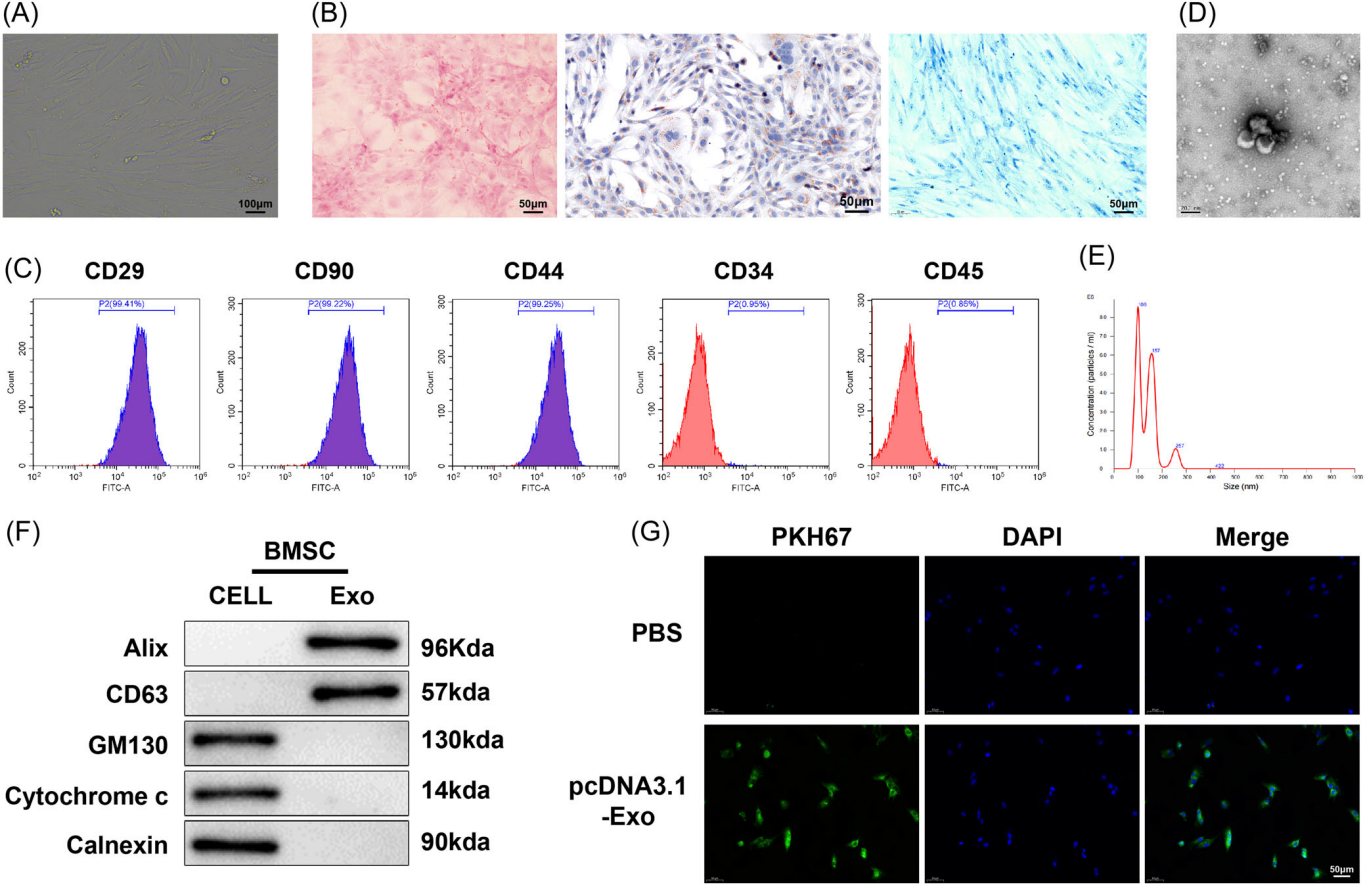
The extraction and identification of BMSCs. (A) Ordinary optical microscope observations of BMSC morphology (×100). (B) Ordinary optical microscope analyses of adipogenic and osteogenic differentiation in BMSCs in vitro (×200). (C) Flow cytometer measurement of surface antigens (positive: CD29, CD90, and CD44; negative: CD45 and CD34). (D) Transmission electron microscope observations of BMSC‐Exos. (E) NTA analyses of BMSC‐Exo size. (F) Western blot examination of the expression of Exo surface marker proteins Alix, CD63, GM130, cytochrome *c*, and calnexin. (G) Laser scanning microscope evaluation of Exo uptake in H9C2 cells. BMSC, bone marrow stromal cells; Exo, exosome; NTA, nanoparticle tracking analysis.

BMSCs were isolated, cultured, and observed under microscopes. The results showed that BMSCs were spindle‐shaped and that the isolated BMSCs exhibited the abilities of osteogenesis, adipogenesis, and chondrogenic differentiation (Figure 1A,B). Flow cytometry detections showed that CD29, CD90, and CD44 had relatively strong positive signals while CD34 and CD45 appeared to be negative (Figure 1C), indicating the successful isolation of BMSCs. Exos were isolated from the BMSC medium and observed to be round or oval membranous vesicles under the transmission electron microscope, and vesicles were surrounded by membranous structures at the periphery, with a low‐density component in the center of the vesicle (Figure 1D). NTA analyses manifested that the particle sizes of major isolated vesicles ranged from 50 to 200 nm (Figure 1E). Western blot results displayed that surface marker proteins Alix and CD63 were expressed but GM130, cytochrome *c*, and calnexin were not expressed in the isolated BMSC‐Exos (Figure 1F). These results validated that the vesicles isolated from BMSCs were Exos. The uptake condition of Exos by H9C2 cells was assessed under LSM with PBS as the control. No signals of green fluorescence were found in the PBS group of H9C2 cells and PHK67‐labeled Exo fluorescence was observed in the Exo group (Figure 1G), which implicated that BMSC‐Exos were internalized by H9C2 cells.

### 3.2 BMSC‐Exos alleviated OS and mitochondrial damage in DOX‐induced H9C2 cells

**Figure 2:**
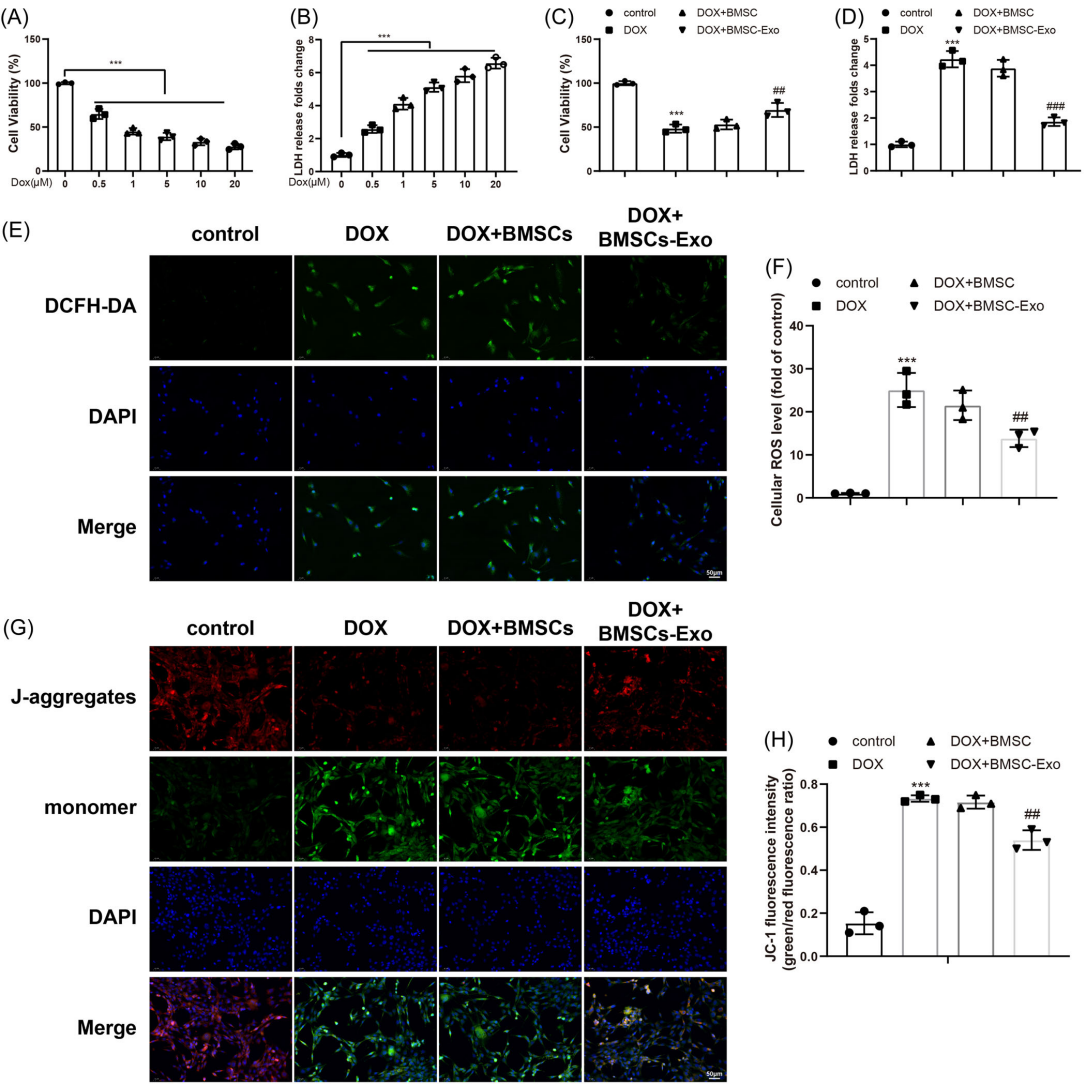
BMSC‐Exos improves OS and mitochondrial damage of DOX‐treated H9C2 cells. (A) CCK‐8 assay to test viability of H9C2 cells 24 h after treatments of different concentrations of DOX (0–20 μM). (B) LDH release of H9C2 cells 24 h after treatment of DOX at above concentrations. (C) CCK‐8 assay to measure changes of cell viability after treatment of 1 μM DOX. (D) LDH release of each group. (E, F) DCFH‐DA staining to test ROS production of cells. (G, H) JC‐1 immunofluorescence to measure the changes of mitochondrial membrane potentials of cells. ***p < .001 versus the control group or the 0 μM group; ^##^ *p* < .01; ^###^ *p* < .001 versus the DOX + BMSC group. One‐way analysis of variance was adopted to confirm *p* values, with Tukey's test for post hoc multiple comparisons. Each experiment was independently repeated thrice. BMSC, bone marrow stromal cells; CCK, cell counting kit; DCFH‐DA, 20,70‐dichlorofluorescein diacetate; DOX, doxorubicin; Exo, exosome; LDH, lactic dehydrogenase; OS, oxidative stress; ROS, reactive oxygen species.

To ascertain whether BMSC‐derived Exos ameliorate DOX‐induced H9C2 cell injury, CCK‐8 and LDH release assays were carried out. The 24‐h treatment of different concentrations of DOX (0–20 μM) dose‐dependently inhibited the viability of H9C2 cells (Figure 2A) and dose‐dependently increased LDH release (Figure 2B). Given these results and previous clinical research,^29^ 1 μM DOX was selected to perform in vitro experiments for the stable construction of the in vitro cardiotoxicity model.

Next, BMSC‐derived Exos and DOX were simultaneously used to treat H9C2 cells. The results of CCK‐8 and LDH release assays indicated that the cell viability was memorably lowered and LDH release was increased in H9C2 cells by DOX treatment, while BMSC‐Exos evidently enhanced cell viability and reduced LDH release in DOX‐treated cells (Figure 2C,D). DCFH‐DA probe examination of ROS contents displayed that single treatment of DOX signally augmented ROS contents in H9C2 cells, whereas treatment of BMSC‐Exos markedly diminished ROS contents in DOX‐treated H9C2 cells. Meanwhile, treatment of BMSCs did not change ROS levels in DOX‐treated H9C2 cells (Figure 2E,F). Additionally, immunofluorescence was adopted to measure the mitochondrial membrane potential of different groups to evaluate the mitochondrial damage. JC‐1 is a fluorescent lipophilic carbonyl blue dye used for the measurement of mitochondrial membrane potential. When the mitochondrial membrane potential is relatively high, JC‐1 converges in matrix to form J‐aggregates, generating red fluorescence (excitation wavelength [Ex]/emission wavelength [Em] = 585/590 nm). When the potential is relatively low, JC‐1 fails to converge in matrix and then exists in the form of monomer, thus producing green fluorescence (Ex/Em = 510/527 nm). In this way, the reduction in mitochondrial membrane potentials can be easily observed through the change of JC‐1 from red fluorescence to green fluorescence. Results reflected that BMSC‐Exos distinctly reversed the DOX‐induced decrease in mitochondrial membrane potentials of H9C2 cells (the ratio of green fluorescence to red fluorescence decreased) (Figure 2G,H). These results indicated that BMSC‐Exos reduced DOX‐induced OS and mitochondrial damage in H9C2 cells.

### 3.3 BMSC‐Exos improved DOX‐induced H9C2 cell pyroptosis

**Figure 3:**
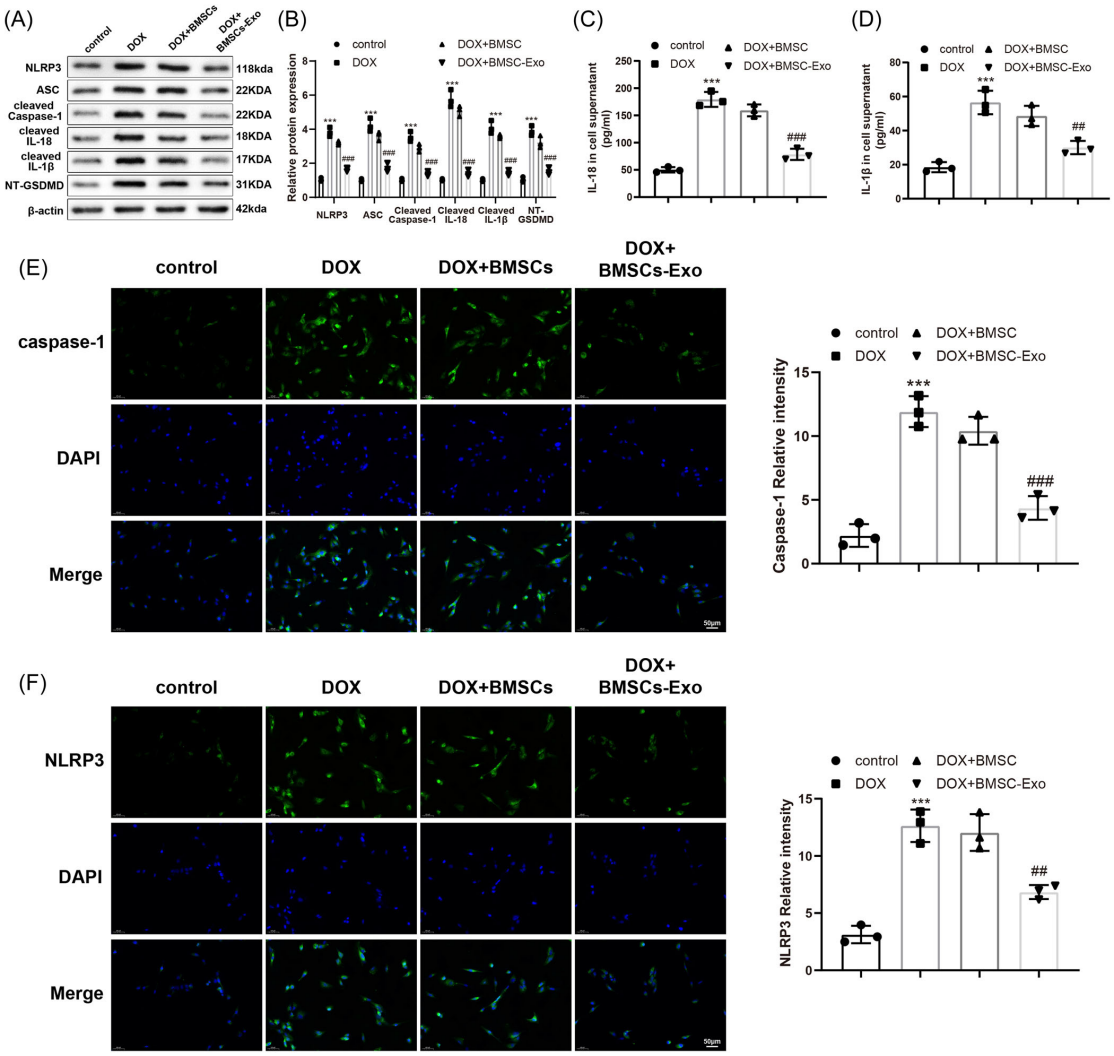
BMSC‐Exos ameliorates DOX‐induced H9C2 cell pyroptosis. (A, B) Western blot detection of the protein expression of NLRP3, ASC, cleaved caspase‐1, cleaved IL‐1β, cleaved IL‐18, and NT‐GSDMD in cells. (C, D) ELISA to test IL‐18 and IL‐1β levels in cell supernatants. (E, F) Immunofluorescence to examine the positive rates of NLRP3 and caspase‐1. ***p < .001 versus the control group; ^##^ *p* < .01; ^###^ *p* < .001 versus the DOX + BMSC group. One‐way analysis of variance was adopted to confirm *p* values, with Tukey's test for post hoc multiple comparisons. Each experiment was independently repeated thrice. ASC, apoptosis‐associated speck‐like protein containing A CARD; BMSC, bone marrow stromal cells; DOX, doxorubicin; ELISA, enzyme‐linked immunosorbent assay; Exo, exosome; IL, interleukin; NLRP3, nucleotide‐binding oligomerization domain, leucine rich repeat and pyrin domain containing 3; NT‐GSDMD, active N‐terminal fragment of gasdermin D.

Whether BMSCs‐derived Exos inhibited pyroptosis in H9C2 cells was further explored in our research. Western blot results displayed that the treatment of DOX markedly induced the activation of NLRP3 and ASC and the cleavage of caspase‐1, GSDMD, IL‐1β, and IL‐18 proteins, which was suppressed by the further addition of BMSC‐Exos (Figure 3A,B). Meanwhile, ELISA detections of IL‐18 and IL‐1β contents in cell supernatants revealed coincident results (Figure 3C,D). Immunofluorescence demonstrated that BMSC‐Exos diminished DOX‐caused elevations in positive rates of NLRP3 and caspase‐1 (Figure 3E,F). The above data indicated that BMSC‐Exos ameliorated DOX‐induced pyroptosis of H9C2 cells.

### 3.4 BMSC‐Exos reduced the transcription of GSDMD via the PI3K‐AKT‐Foxo1 axis in DOX‐treated H9C2 cells

**Figure 4:**
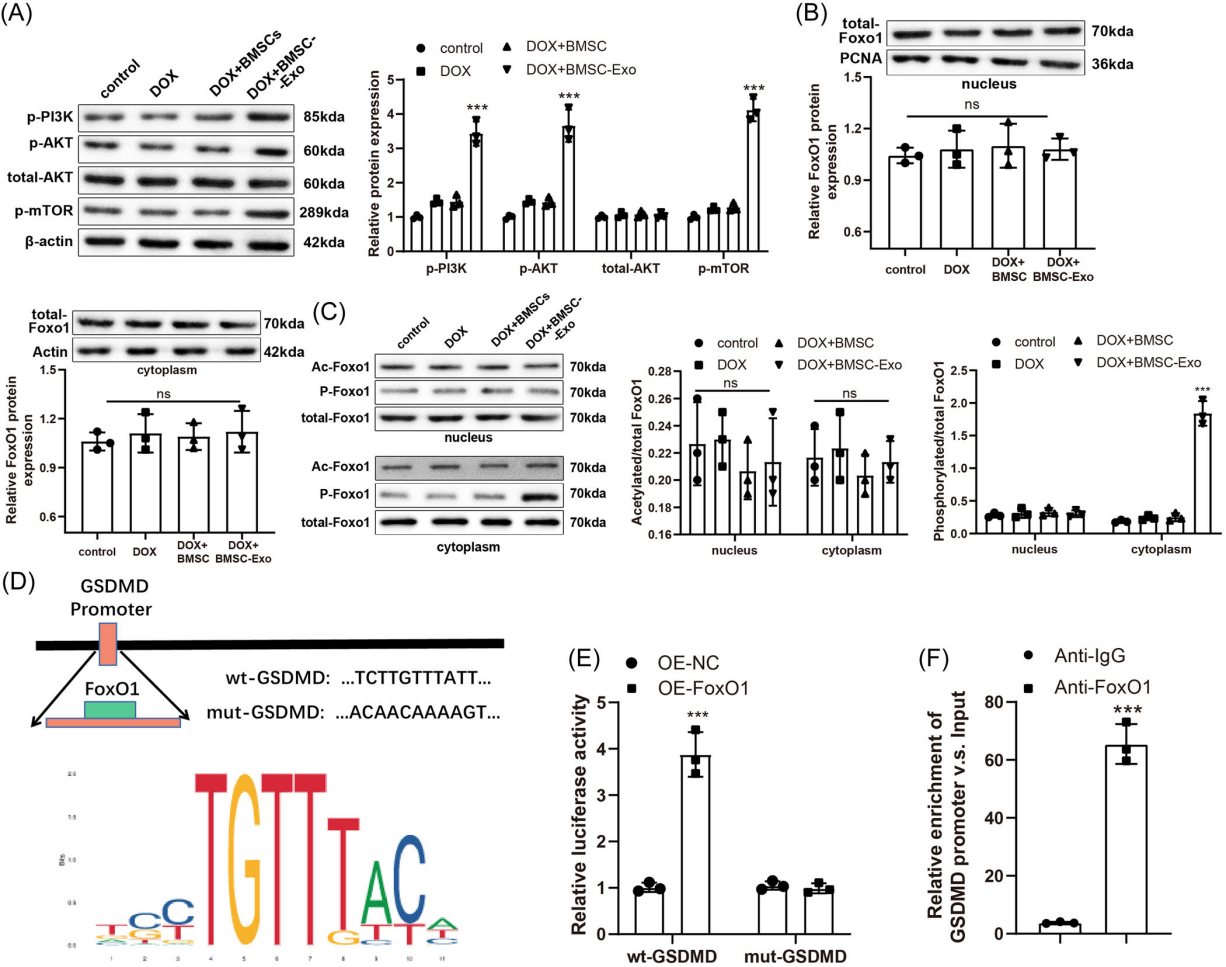
BMSC‐Exos regulates GSDMD transcription through the PI3K‐AKT‐Foxo1 pathway in DOX‐induced H9C2 cells. (A) Western blot to examine the expression of p‐PI3K, p‐AKT, total‐AKT, and p‐mTOR in cells. *p < .05; **p < .01; ***p < .001 versus the DOX + BMSC group. (B) Western blot to measure the protein expression levels of total‐Foxo1 in cell nucleus and cytoplasm. (C) Western blot to determine the expression of acetylated Foxo1, phosphorylated Foxo1, and total‐Foxo1,  ***p < .001 versus the DOX + BMSC group. (D) Public database to analyze the binding site sequence of Foxo1 to GSDMD. (E) Dual‐luciferase reporter gene assay to validate binding of Foxo1 to GSDMD promoter,  ***p < .001 versus the OE‐NC group. (F) ChIP assay to test the enrichment level of Foxo1 in the promoter region of GSDMD, ***p < .001 versus the anti‐IgG group. The two‐tailed test was employed to confirm *p* values. Except for special statements, one‐way analysis of variance was adopted to confirm *p* values, with Tukey's test for post hoc multiple comparisons. Each experiment was independently repeated thrice. AKT, protein kinase B; BMSC, bone marrow stromal cells; ChIP, chromatin immunoprecipitation; DOX, doxorubicin; Exo, exosome; GSDMD, gasdermin D; IgG, immunoglobulin G; mTOR, mechanistic target of rapamycin kinase; OS, oxidative stress; p, phosphorylated; PI3K, phosphatidylinositol 3‐kinase.

Next, we explored the mechanism of BMSC‐Exos in DOX‐induced myocardial injury. Western blot results manifested that the treatment of BMSC‐Exos conspicuously increased the levels of p‐PI3K, p‐AKT, and p‐mTOR in DOX‐treated H9C2 cells (Figure 4A). In addition, western blot data uncovered insignificant differences in total protein expression of Foxo1 in both cytoplasm and nucleus (Figure 4B), and acetylated Foxo1 expression exhibited a similar condition (Figure 4C). However, BMSC‐Exos treatment evidently enhanced the levels of phosphorylated Foxo1 in the cytoplasm of DOX‐treated H9C2 cells, accompanied by no significant difference in the levels of phosphorylated Foxo1 in the nucleus (Figure 4C). These findings illustrated BMSC‐Exos might phosphorylate Foxo1 through the PI3K‐AKT pathway to promote the translocation of Foxo1 from the nucleus to the cytoplasm and inactivate its transcription, thus suppressing Foxo1‐modulated downstream gene expression and further regulating OS and pyroptosis in H9C2 cells. In addition, it was found via a public database that Foxo1, as a transcription factor, regulated the transcription of GSDMD (Figure 4D). The dual‐luciferase reporter gene assay unveiled that OE‐Foxo1 treatment prominently elevated the luciferase activity of 293T cells transfected with wt‐GSDMD promoter sequence‐inserted reporter vectors but did not alter the luciferase activity of 293T cells transfected with mut‐GSDMD promoter sequence‐inserted reporter vectors (Figure 4E). ChIP assay results showed that Foxo1 remarkably enriched in the promoter region of GSDMD (Figure 4F). Conclusively, BMSC‐Exos repressed GSDMD transcription through the PI3K‐AKT‐Foxo1 axis in DOX‐treated H9C2 cells.

### 3.5 Inhibition of the PI3K‐AKT pathway by API‐2 nullified the alleviatory impact of BMSC‐Exos on DOX‐induced OS and pyroptosis in H9C2 cells

**Figure 5:**
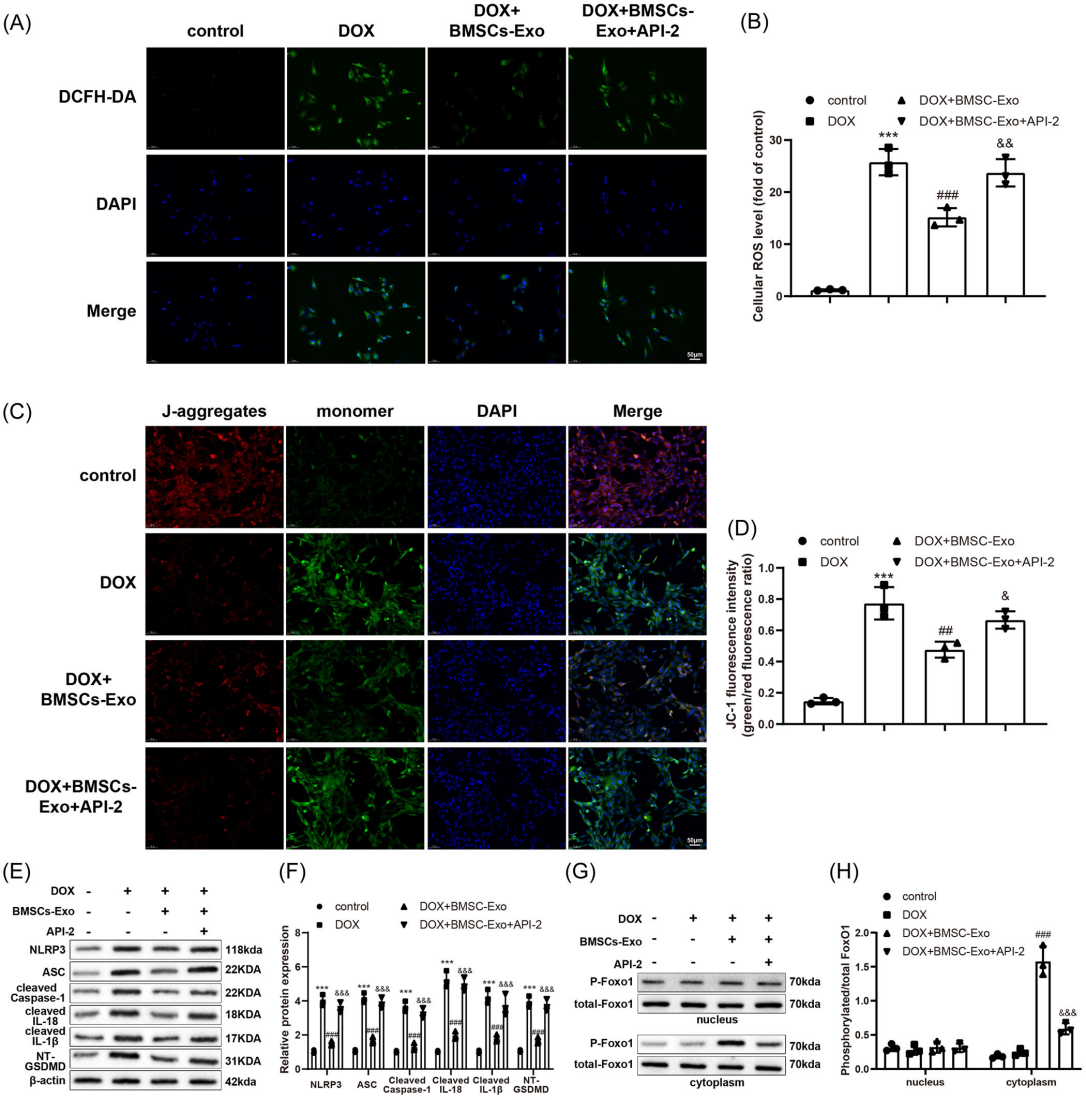
Inactivation of the PI3K‐AKT pathway abrogates the relieving impacts of BMSC‐Exos on DOX‐induced OS and pyroptosis in H9C2 cells. (A, B) DCFH‐DA staining to measure ROS levels in cells. (C, D) JC‐1 immunofluorescence to monitor the changes of potential difference of mitochondrial membrane in cells. (E, F) Western blot to test the expression of NLRP3, ASC, cleaved caspase‐1, NT‐GSDMD, cleaved IL‐1β, and cleaved IL‐18 in cells. (G, H) Western blot to measure the phosphorylation levels and total protein levels of Foxo1 in cell nucleus and cytoplasm; ***p < .001 versus the control group; ^##^ *p* < .01; ^###^ *p* < .001 versus the DOX group; ^&^ *p* < .05; ^&&^ *p* < .01; ^&&&^ *p* < .001 versus the DOX + BMSC‐Exo group. One‐way analysis of variance was adopted to confirm *p* values, with Tukey's test for post hoc multiple comparisons. Each experiment was independently repeated thrice. AKT, protein kinase B; ASC, apoptosis‐associated speck‐like protein containing A CARD; BMSC, bone marrow stromal cells; DCFH‐DA, 20,70‐dichlorofluorescein diacetate; DOX, doxorubicin; Exo, exosome; Foxo1, forkhead box O1; GSDMD, gasdermin D; IL, interleukin; NLRP3, nucleotide‐binding oligomerization domain, leucine rich repeat and pyrin domain containing 3; OS, oxidative stress; PI3K, phosphatidylinositol 3‐kinase; ROS, reactive oxygen species.

To further clarify whether BMSC‐Exos regulated GSDMD transcription via the PI3K‐AKT‐Foxo1 to improve OS and pyroptosis in DOX‐induced myocardial injury, the selective inhibitor of AKT, API‐2, was chosen to treat H9C2 cells after Exo treatment. DCFH‐DA staining exhibited that the ROS level was enormously augmented in the DOX + BMSC‐Exo + API‐2 group versus the DOX + BMSC‐Exo group (Figure 5A,B). JC‐1 immunofluorescence demonstrated that in contrast to the DOX + BMSC‐Exo group, the potential difference of mitochondrial membrane was observably lowered in the DOX + BMSC‐Exo + API‐2 group (the ratio of green fluorescence to red fluorescence was enhanced) (Figure 5C,D). Meanwhile, western blot results revealed that the activation of NLRP3 and ASC and in vitro cleavage of caspase‐1, GSDMD, IL‐1β, and IL‐18 proteins distinctly increased in the DOX + BMSC‐Exo + API‐2 group in comparison with the DOX + BMSC‐Exo group (Figure 5E,F). Western blot showed that API‐2 markedly decreased the phosphorylation levels of Foxo1 in the cytoplasm of H9C2 cells (Figure 5G,H). In summary, API‐2 blocked the improving effect of BMSC‐Exos on DOX‐induced OS and pyroptosis in H9C2 cells.

## 4 DISCUSSION

DOX causes myocardial injury, to which OS and pyroptosis were closely linked.^30^, ^31^ Recent research revealed that MSC‐derived Exos protect against myocardial infarction and myocardial ischemia–reperfusion injury, so increasing attention is paid to their potential therapeutic values in treating myocardial problems.^32^, ^33^ To probe the specific role of BMSC‐Exos in DOX cardiotoxicity, this study was conducted to ascertain the function of BMSC‐Exos in OS and pyroptosis during DOX‐induced myocardial injury and the related mechanism. It was elucidated from our data that BMSC‐Exos ameliorated OS and pyroptosis in DOX‐induced myocardial injury via the PI3K‐AKT‐Foxo1‐GSDMD axis.

BMSC‐Exos is implicated in the suppression of DOX‐induced cardiotoxicity.^34^ Therefore, to delve into the specific role of BMSC‐Exos in DOX cardiotoxicity, Exos were isolated from rat BMSCs, and the following experiments were conducted. LDH is an enzyme that plays a part in myocardial ischemia–reperfusion injury.^35^, ^36^ ROS is a product in the pro‐/antioxidant balance and contributes to harmful OS when excessively generated, the clearance of which is crucial for the improvement of myocardial injury.^37^, ^38^ JC‐1 is a cationic fluorescent dye to assess the mitochondrial membrane potential that reflects the cytotoxicity when lost.^39^, ^40^ Therefore, these three factors were selected for the detection of OS and mitochondrial damage, along with the measurement of cell viability. The results elucidated that BMSC‐Exo treatment elevated viability and red‐to‐green fluorescence ratio of JC‐1 and downregulated ROS levels in DOX‐treated H9C2 cells. Intriguingly, a former study showed accordant trends that addition of BMSC‐Exos increased proliferation and mitochondrial potential and decreased ROS levels in hypoxia/reoxygenation‐treated myocardial cells.^41^ Meanwhile, NLRP3 is an inflammasome participating in cell pyroptosis.^42^ ASC is an adaptor protein required for driving pyroptosis.^43^ Caspase‐1 is an inflammatory mediated enzyme, which cleaves and activates inflammatory factors.^44^ Along with the inflammatory factors IL‐1β and IL‐18, caspase‐1 contributes to pyroptosis.^45^, ^46^, ^47^ Therefore, the aforementioned factors were all tested in our experiments to evaluate pyroptosis during DOX‐induced myocardial injury. Earlier research discovered that BMSC‐Exos reduced the level of IL‐1β in H_2_O_2_‐treated H9c2 cells,^48^ which was concurrent with our results. Additionally, we also observed that BMSC‐Exos suppressed the activation of NLRP3 and ASC and the expression of caspase‐1, GSDMD, and IL‐18. Zeng et al. also concluded that BMSC‐Exos reduced pyroptosis in cerebral ischemia–reperfusion injury.^49^

It has been documented that the PI3K‐AKT pathway plays a vital role in protecting against myocardial injury.^50^ Furthermore, DOX blocked the PI3K‐AKT‐mTOR pathway to induce myocardial injury.^51^ In addition, the PI3K‐AKT pathway activation is involved in the alleviation of OS‐induced apoptosis and ischemia/reperfusion‐induced pyroptosis.^52^, ^53^ Of note, BMSC‐Exos has been validated to activate the PI3K‐AKT pathway to reduce myocardial ischemia–reperfusion injury.^54^ Concordantly, our data also uncovered that BMSC‐Exo treatment increased the levels of p‐PI3K and p‐AKT in cells and p‐Foxo1 in the cytoplasm of cells. Previous documentation concluded that posttranslational modifications of Foxo1 are related to myocardial ischemia^55^ and that the knockdown of Foxo1 attenuates DOX‐induced myocardial injury.^56^ In addition, Foxo1 can be phosphorylated by AKT, resulting in the translocation of Foxo1 from the nucleus to the cytoplasm.^57^ Additionally, a previous study revealed that Foxo1 can bind to GSDMD promoter during myocardial damage.^58^ Of note, our observations also verified the binding relationship between Foxo1 and GSDMD. More importantly, GSDMD is an important pyroptosis mediator and DOX directly binds to GSDMD to promote pyroptosis of myocardial cells.^27^ In addition, GSDMD axis is deeply associated with OS and pyroptosis of myocardial cells in myocardial infarction.^59^ These discussions confirmed the linkages among PI3K‐AKT, Foxo1, and GSDMD in DOX‐induced myocardial injury and implicated their involvement in the alleviatory effects of BMSC‐Exo on DOX‐induced myocardial injury. Therefore, the inhibitor of AKT, API‐2,^60^ was further used in our research for assessing the effect of this pathway on OS and pyroptosis in DOX‐induced myocardial injury. Our data unraveled that API‐2 exaggerated OS and pyroptosis mitigated by BMSC‐Exos in DOX‐induced myocardial injury. To be specific, API‐2 treatment elevated ROS generation, lowered the mitochondrial membrane potential, and enhanced the protein levels of cleavage‐caspase‐1, GSDMD, IL‐1β, and IL‐18 in H9C2 treated with DOX + BMSC‐Exos, accompanied by the diminished phosphorylated levels of Foxo1 in the cytoplasm.

In a word, it can be implicated from our results that BMSC‐Exos improved OS and pyroptosis in DOX‐induced myocardial injury by downregulating GSDMD via the PI3K‐AKT‐Foxo1 pathway. However, we only probed this mechanism in the cellular model. Further related animal and clinical studies are warranted to be carried out to further validate our findings. Nevertheless, our experiment results might assist in providing data for promoting investigations on the therapeutic potential of BMSC‐Exos in myocardial injury.

## AUTHOR CONTRIBUTIONS

SL, ZH, YY and TFF conceived the ideas; designed the experiments. ZH, YY, TFF, ZYL and LST performed the experiments. ZH, YY, TFF, ZPT and CYM analysed the data. ZYL and LST provided critical materials. ZPT and CYM wrote the manuscript. SL supervised the study. All the authors have read and approved the final version for publication.

## CONFLICT OF INTEREST STATEMENT

The authors declare no conflict of interest.

## Data Availability

The data sets used or analyzed during the current study are available from the corresponding author on reasonable request.
